# CONNECT4 *APOE*: A randomized trial of telephone versus real‐time two‐way videoconference for disclosure of *APOE* genotype results in cognitively unimpaired adults

**DOI:** 10.1002/alz.71658

**Published:** 2026-07-27

**Authors:** Elisabeth McCarty Wood, Jessica B. Langbaum, Brian L. Egleston, Demetrios Ofidis, Cara Cacioppo, Carolyn M. Langlois, Michelle Weinberg, Scott Y. H. Kim, Eric M. Reiman, Pierre N. Tariot, J. Scott Roberts, Jason Karlawish, Angela R. Bradbury

**Affiliations:** ^1^ Department of Medicine Division of Hematology‐Oncology University of Pennsylvania Perelman School of Medicine Philadelphia Pennsylvania USA; ^2^ Banner Alzheimer's Institute Phoenix Arizona USA; ^3^ Fox Chase Cancer Center Temple University Philadelphia Pennsylvania USA; ^4^ Department of Bioethics NIH Clinical Center Bethesda Maryland USA; ^5^ Department of Health Behavior and Health Equity University of Michigan School of Public Health Ann Arbor Michigan USA; ^6^ Department of Medical Ethics and Health Policy, Perelman School of Medicine University of Pennsylvania Philadelphia Pennsylvania USA

**Keywords:** *APOE*, genetic counseling, genetic testing, telehealth

## Abstract

**INTRODUCTION:**

Data are needed to inform best practices for disclosing apolipoprotein E (APOE) results given increasing indications for testing.

**METHODS:**

CONNECT4, a multi‐center, randomized trial (NCT02978729) compared disclosure of *APOE* genotype by videoconference or telephone among cognitively unimpaired adults 60–75 years of age. Outcomes were collected at baseline, 2–7 days, 6 weeks, and 6 months.

**RESULTS:**

A total of 301 participants were randomized to telephone and 297 to videoconference disclosure. There were no significant differences between arms for primary or secondary patient‐reported outcomes. Moderator analyses found that videoconference reduced depressive symptoms more for older participants (70–75 year), whereas teleconference reduced depressive symptoms more for younger participants (60–64 years) and videoconference increased knowledge for non‐White participants as compared to White participants.

**DISCUSSION:**

These data suggest that both telephone and videoconference are reasonable options for disclosure of *APOE* genotype in cognitively unimpaired individuals. Videoconference disclosure may have some benefits for certain demographic groups.

## Highlights

- Randomized trial of genetic result disclosure via telehealth modalities
- Study participants received genetic counseling for apolipoprotein E (*APOE*) genotype results
- Findings support both telephone and videoconference for *APOE* result disclosure
- Future importance as *APOE* genetic testing demand increases for Alzheimer's disease

## 1 BACKGROUND

Apolipoprotein E (*APOE*) testing identifies individuals at increased risk for late‐onset Alzheimer's disease (AD) and is increasingly incorporated into research and clinical care.^1^, ^2^ Preclinical AD trials may target or restrict enrollment to *APOE* ε4 carriers, who have the highest lifetime AD risk. With U.S. Food and Drug Administration (FDA)–approved anti‐amyloid therapies, and given the heightened risk of amyloid‐related imaging abnormalities (ARIA) in ε4 carriers, *APOE* testing is now recommended before treating mild cognitive impairment (MCI) and mild dementia due to AD.^3^ This is expected to increase interest in presymptomatic testing among relatives of *APOE* ε4 carriers screened for therapy.^1^, ^2^

For these reasons, effective communication of *APOE* results has become essential. The Risk Evaluation and Education for Alzheimer's Disease (REVEAL) studies demonstrated that *APOE* disclosure generally did not cause clinically significant psychological harm, although carriers reported greater test‐specific distress than non‐carriers.^4^ REVEAL2 showed that a brief educational brochure was non‐inferior to extensive counseling when paired with genetic counselor–led sample collection and results disclosure.^5^ REVEAL3 found that telephone disclosure was non‐inferior to in‐person disclosure for non‐carriers but lacked sufficient ε4 carrier data to draw firm conclusions for that group.^6^ Limitations of these studies included small samples of ε4 carriers, especially homozygotes; higher education; and participants often being younger than is typical for clinical manifestation of AD. Videoconferencing for disclosure of *APOE* results was also found to be an effective alternative to in‐person disclosure in a randomized study of 93 first‐degree relatives of patients with AD.^7^

The Alzheimer's Prevention Initiative (API) Generation Study 1 (GS1) was the first National Institutes of Health (NIH) and industry‐supported AD prevention trial targeting cognitively unimpaired *APOE* ε4 homozygotes.^8^, ^9^, ^10^, ^11^ GS1 used a one‐visit genetic counseling model, using pre‐test education via video and/or brochure followed by in‐person results disclosure, as described previously in Langlois et al.^12^ Due to the limited availability of trained providers, many sites partnered with the Penn Telegenetics Program for remote disclosure of results by a genetic counselor (GC). Although prior studies in cancer genetics established that genetic counseling by phone is non‐inferior to in‐person counseling,^13^, ^14^, ^15^ few studies have compared remote phone counseling to videoconferencing.^16^ To address these gaps, we developed CONNECT4 (Remote COmmuNication of GeNEtiC Test Results for *APOE*), a companion study randomizing participants to phone versus videoconference disclosure. We hypothesized that videoconferencing would improve short‐ and long‐term cognitive and emotional responses. We also aimed to explore whether outcomes differ by disclosure method and *APOE* genotype (homozygote, heterozygote, non‐carrier) and identify patient factors that influence disclosure impact.

## 2 METHODS

### 2.1 Study design, setting, and participants

From August 2016 to July 2019, participants from 20 GS1 sites within the United States (13 states) were recruited to the CONNECT4 Study (NCT02978729) comparing disclosure of *APOE* ε4 genotype results by remote videoconference or telephone with a GC in the Penn Telegenetics Program. Data release for both GS1 and CONNECT4 to the United States was delayed due to the implementation of European Union General Data Protection Regulation rules.^17^ The University of Pennsylvania Institutional Review Board approved this study. Data collection for GS1 was terminated early in July 2019 due to safety concerns with umibecestat. This led to an early termination of CONNECT4 as well.

Participants in GS1 were 60–75 years old, cognitively unimpaired adults identified through pre‐existing registries and databases at local sites and by individuals who self‐referred after learning about the trial.^8^ Cognitive status and psychological readiness for disclosure were assessed per the GS1 protocol, using the Mini‐Mental State Examination (MMSE; ≥24), Geriatric Depression Scale (GDS; score ≤6; scores between 7 and 10 included based on local site investigator judgment) and Modified State Trait Anxiety Inventory (score ≤17; scores of 18 or 19 included based on investigator judgment).^18^ Participants were offered enrollment in CONNECT4 at the time of their *APOE* disclosure visit for GS1. If they declined, they received their results by telephone with a Penn Telegenetics GC according to the GS1 protocol. Participants interested in CONNECT4 were contacted by Penn Telegenetics and provided verbal consent to CONNECT4.

*APOE* testing was conducted according to the GS1 protocol, as described previously.^12^ Both participants receiving first‐time disclosure of their *APOE* genotype as well as those with prior knowledge of their genotype from previous clinical, research, or direct‐to‐consumer (DTC) testing were eligible for CONNECT4. If previous testing was performed in a non‐CLIA (Clinical Laboratory Improvement Amendments) research or DTC laboratory, CLIA confirmation of *APOE* genotype was required prior to the disclosure visit, per the GS1 protocol. Participants with prior knowledge were included as most had not had genetic counseling, and information on risks for AD based on *APOE* genotype could have changed since they received their results.

**RESEARCH IN CONTEXT**

1. **Systematic review**: A literature review was conducted using traditional sources regarding communication of apolipoprotein E (*APOE*) genotype results for Alzheimer's disease (AD) risk assessment, as well as genetic counseling via telehealth. These findings informed a companion study to an AD prevention clinical trial in which participants were randomized to telephone versus videoconference for *APOE* genotype disclosure.
2. **Interpretation**: The data suggest that both telephone and videoconference are reasonable options for disclosure of *APOE* results in cognitively unimpaired individuals. Videoconference disclosure may reduce depressive symptoms and have greater knowledge gains in some subgroups.
3. **Future directions**: These findings are especially relevant as demand for *APOE* genotyping has increased due to *ε4* status informing risk of amyloid‐related imaging abnormalities (ARIA) during treatment with anti‐amyloid therapy. Future studies may contribute to real‐world data regarding the use of telehealth to support not only AD clinical populations but also their family members interested in genetic testing for AD.

### 2.2 Randomization

Participants were assigned equally to videoconference or telephone disclosure, stratified by site and biological sex, using a permuted block design. Participants were informed of their randomization arm after completion of their baseline survey (T0).

### 2.3 Procedures

The API Genetic Counseling and Disclosure Process uses a condensed model that can be completed in a single visit. An Educational Pre‐Disclosure Video (Table S1), which covered information typically addressed in a pre‐testing genetic counseling session, was available to all participants.^12^ *APOE* disclosure visit materials included talking points, visual aids, and result summary sheets designed for GS1. For the CONNECT4 study, standardized communication protocols for telephone and videoconference were utilized and adapted for *APOE* result disclosure.^19^ The 10 participating GCs completed protocol and technology training; all GCs performed both telephone and videoconference disclosures. GCs completed a sessions checklist (Figure S1) and all sessions were audiotaped for fidelity assessments.

Telephone disclosure (TD) sessions were scheduled and conducted at study sites in a private room with a private telephone line. Real‐time two‐way videoconference disclosure (VCD) sessions were scheduled and conducted at a study site in a private room. VCD sessions were conducted on study laptops with web cameras and secure teleconferencing software (BlueJeans, Medisprout). When technology challenges occurred (e.g., failure to connect, maintain a connection), GCs made three attempts to reconnect and if unsuccessful the session was converted to telephone. Study assessments were collected as part of the GS1 protocol by trained personnel by telephone at 2–7 days (T1), 6 weeks (T2), and 6 months (T3) in participants who completed their *APOE* disclosure.

### 2.4 Primary outcomes

Outcomes were based on[Fig alz71658-fig-0001] our theoretical model informed by the Self‐regulation Theory of Health Behavior,^20^, ^21^, ^22^, ^23^ including potential risks of telephone and videoconference communication, (e.g., poorer understanding of results, greater short‐term distress, and poorer behavioral outcomes), and as described previously.^21^

*Knowledge of genetic disease* (T0–T3) was assessed using a 10‐item scale adapted to evaluate genetic knowledge in the context of *APOE* testing for AD, with total scores ranging from 10 to 50, with higher scores indicating greater knowledge (Cronbach's α = 0.59‐0.65).^24^, ^25^

*Disease‐specific distress (DSD, T0*–*T3)* was evaluated using an 8‐item questionnaire adapted from the Impact of Events Scale (IES), consistent with studies evaluating outcomes of genetic testing where the scale is a measure of distress specific to a condition or disease risk.^26^, ^27^, ^28^, ^29^ Here the stressor is being at risk for a disease (e.g., “I thought about Alzheimer's disease when I didn't mean to.”) and is measured at baseline to understand how the impact of genetic testing or genotype disclosure might change this stress response. Scores range from 0 to 40, with higher scores indicating greater levels of distress (Cronbach's α = 0.79‐0.82).^30^

*Satisfaction with Remote Counseling* (T1) was evaluated using 10 items adapted for genetic counseling to evaluate patient perceived provider comfort, patient satisfaction with privacy and patient comfort with utilized audio/visual technology (Cronbach's α = 0.74).^20^, ^31^ Scores range from 10 to 50, with higher scores indicating greater level of satisfaction.

### 2.5 Secondary outcomes

*Depressive symptoms* (T0–T3) were assessed with the 15‐item version of the GDS (Cronbach's α = 0.56–0.75). Scores range from 0 to 15, with scores 5 or above indicating symptoms of potential[Table alz71658-tbl-0001], [Table alz71658-tbl-0002], [Table alz71658-tbl-0003], [Table alz71658-tbl-0004], [Table alz71658-tbl-0005], [Table alz71658-tbl-0006] clinical concern.^32^

*Test related distress* (T1–T3) was measured using the Impact of Genetic Testing in Alzheimer's Disease Scale (IGT‐AD), a 16‐item measure consisting of two subscales: a 12‐item distress subscale to assess psychological distress in response to learning test results (subscale range 0–60; higher scores indicate greater distress), and a 4‐item positive response subscale to assess psychological benefits (e.g., relief) to learning test results (subscale range 0–20; lower scores indicate greater positive feelings).^33^ Participants could respond “never,” “rarely,” “sometimes,” or “often” to questions such as “Feeling upset about my test result,” with the responses converted to a 0–5 scale. Responses to items are summed to provide a total IGT‐AD score, ranging from 0 to 80. Higher scores for the overall score and both the distress and positive subscales indicate greater levels of distress or less positive responses (Cronbach's α = 0.6–0.72).

*Perceived risk of AD* (T0–T3) was measured with selected items used or adapted from the REVEAL Study to assess participants’ beliefs regarding their likelihood on a scale of 0–100% of developing AD in the future.^34^ These included reporting a numerical lifetime risk of AD (0–100%), perceived risk of AD compared to others of the same age and gender (5‐point Likert scale from much lower to much higher), and perceived lifetime risk of AD (5‐point Likert scale from very low to very high).

*Perceived threat of AD* (T0–T3) was measured with a 7‐item scale assessing participants’ level of worry about developing AD and their beliefs about its likelihood and severity. Scores range from 0 to 35, with higher scores indicating greater levels of perceived threat (Cronbach's α = 0.54–0.56).^35^

*Satisfaction with genetic services* (T1) was assessed with a 9‐item satisfaction with communication scale, which evaluates participants’ perceptions of their genetic counseling and testing experience (Cronbach's α = 0.79).^20^, ^36^ Responses are collected on a 5‐point Likert type scale, with higher scores indicating greater level of satisfaction.

### 2.6 Statistical analysis

Our three primary endpoints were change from baseline to 2–7 days post disclosure in: (1) genetic knowledge, (2) disease‐specific distress, and (3) cross‐sectional satisfaction in remote services after genotype disclosure. We used *t*‐tests and Fisher's exact tests for comparisons of arms and genotype groups. Our primary analysis was an intention‐to‐treat analysis. Regression models with interaction terms and controlling for genotype were used to investigate moderators of the randomization arm effects. For the intention‐to‐treat, interaction, and genotype comparisons of patient reported outcomes, we used the multiple imputation techniques of Raghunathan and colleagues to account for missing data.^37^ Based on preliminary differences observed in our previous studies, we anticipated 85% power and 1.67% Type I error (two‐sided) with as few as 342 evaluable subjects/arm (e.g., prior knowledge change score differences of 0.20, standard deviation [SD] = 2.64 versus –0.48 [SD 2.54] between arms). Nominal *p*‐values < 0.0167 defined statistical significance for comparisons of patient reported outcomes between randomization arms (including comparisons by genotype), whereas *p* < 0.05 defined statistical significance for secondary exploratory analyses such as comparisons by technical difficulty status or moderation investigations. The full protocol analytic plan can be found in Supplemental Document 1.

## 3 RESULTS

### 3.1 Study participants

**FIGURE 1 alz71658-fig-0001:**
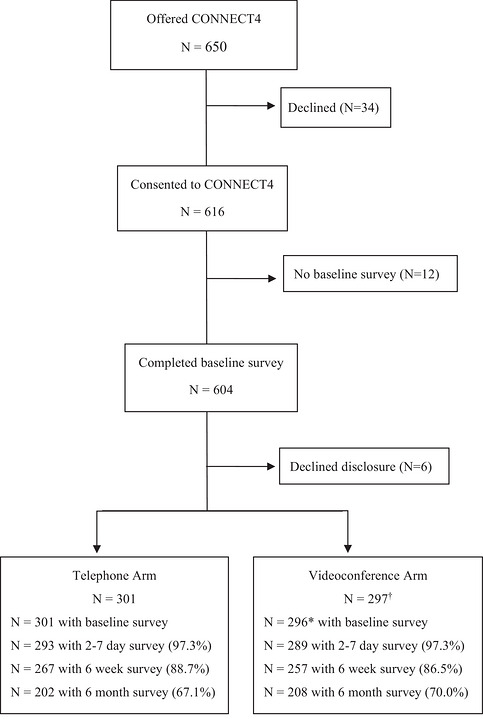
Consort diagram. *One participant without baseline PROs had subsequent PROs data available for the 2–7 day survey. ^†^18 participants experienced technology difficulties that resulted in disclosure completion via telephone.

Enrollment, randomization, and survey completion data are shown in Figure 1. Six hundred sixteen (94.8%) individuals consented to the study and 604 (98%) completed the baseline survey and were randomized, and 598 of these (99%) chose to have their *APOE* genotype disclosed. Those who declined participation (*n* = 34) had neither statistically significant nor clinically relevant differences from those who consented with respect to depressive symptoms, MMSE score, state anxiety, age, race, Hispanic ethnicity, or *APOE* genotype.

Of those choosing disclosure, 301 participants were randomized to TD and 297 to VCD. There were no statistically significant baseline differences between randomized groups (Table 1). The mean age was 67.6 years and the mean years of education was 16.5 (i.e., college graduates); 62.2% of participants were women, 93.3% White and 80.1% reported relatives with a history of AD. One hundred thirty (21.7%) were *APOE* ε4 homozygotes, 307 (51.3%) were heterozygotes, and 161 (26.9%) were non‐carriers.

**TABLE 1 alz71658-tbl-0001:** Baseline characteristics by randomization arm.

	Randomization arm	
	Telephone	Videoconference	Total	
	(*N* = 301)	(*N* = 297)	(*N* = 598)	*p*‐value
**Age**				0.860
Mean (SD)	67.55 (4.33)	67.62 (4.18)	67.59 (4.26)	
Min, max	60.0, 75.0	60.0, 75.0	60.0, 75.0	
**Sex**				0.613
Male	117 (38.9%)	109 (36.7%)	226 (37.8%)	
Female	184 (61.1%)	188 (63.3%)	372 (62.2%)	
**Race**				1.000
Black	5 (1.7%)	5 (1.7%)	10 (1.7%)	
White	280 (93.3%)	277 (93.3%)	557 (93.3%)	
Other	15 (5.0%)	15 (5.1%)	30 (5.0%)	
**Ethnicity**				0.304
Hispanic or Latino	9 (3.0%)	6 (2.0%)	15 (2.5%)	
Not Hispanic or Latino	276 (92.0%)	268 (90.2%)	544 (91.1%)	
Unknown or not reported	15 (5.0%)	23 (7.7%)	38 (6.4%)	
**Years of education**				0.733
Mean (SD)	16.54 (2.80)	16.46 (2.84)	16.50 (2.82)	
Min, max	0.0, 25.0	1.0, 25.0	0.0, 25.0	
**Number of FDR/SDR with AD**				0.579
Mean (SD)	2.07 (1.65)	1.99 (1.72)	2.03 (1.68)	
Min, max	0.0, 10.0	0.0, 9.0	0.0, 10.0	
***APOE* ε4 carrier status**				0.107
Homozygote	65 (21.6%)	65 (21.9%)	130 (21.7%)	
Heterozygote	144 (47.8%)	163 (54.9%)	307 (51.3%)	
Non‐carrier	92 (30.6%)	69 (23.2%)	161 (26.9%)	
**Prior knowledge of *APOE* genotype**				0.722
No	278 (93.9%)	271 (94.8%)	549 (94.3%)	
Yes	18 (6.1%)	15 (5.2%)	33 (5.7%)	
**Viewed educational video**				0.589
No	28 (9.3%)	35 (11.8%)	63 (10.6%)	
Yes	273 (90.7%)	261 (88.2%)	534 (89.4%)	
**Disclosure session length (minutes)**				0.420
Mean (SD)	58.58 (13.70)	59.50 (14.13)	59.04 (13.91)	
Min, max	18, 112	26, 120	18, 120	
**Participant accompanied by family member/friend**				0.189
No	211 (71.3%)	221 (76.2%)	432 (73.7%)	
Yes	85 (28.7%)	69 (23.8%)	154 (26.3%)	
**Technology issues impacted session initiation**				<0.001
No	292 (97.0%)	242 (81.5%)	534 (89.3%)	
Yes	9 (3.0%)	55 (18.5%)	64 (10.7%)	

Abbreviations: AD, Alzheimer's disease; FDR, first‐degree relative; SDR, second‐degree relative.

### 3.2 Differences in cognitive and affective outcomes between randomized arms

There were no differences in primary outcomes (knowledge, disease‐specific distress, and satisfaction with remote services) between arms (Table 2). There were no differences in the change in secondary affective outcomes between randomized arms. The one exception to this was higher IGT‐AD positive response subscale (demonstrating less positive feelings) at 2–7 days after disclosure in the videoconference arm, although this difference between arms did not persist over time.

**TABLE 2 alz71658-tbl-0002:** Differences in cognitive and affective outcomes by randomized arms.

	Randomization arm	
	Telephone	Videoconference	
	(*N* = 301); Mean (SD)	(*N* = 297); Mean (SD)	*p*‐value
**Knowledge of genetic disease** ^a^			
Baseline (pre‐disclosure)	37.41 (4.07)	37.30 (4.21)	0.741
2–7 day change	1.52 (3.75)	1.57 (3.79)	0.864
6‐week change	1.26 (3.73)	1.56 (4.28)	0.393
6‐month change	1.02 (4.07)	1.11 (4.67)	0.806
**Disease‐specific distress** ^a^			
Baseline (pre‐disclosure)	4.66 (5.65)	4.09 (5.18)	0.195
2–7 day change	0.23 (5.03)	1.00 (5.40)	0.076
6‐week change	−0.10 (5.90)	0.30 (5.13)	0.396
6‐month change	0.52 (6.08)	0.90 (5.85)	0.468
**Satisfaction with remote counseling** ^a^			
2–7 days	44.64 (5.00)	44.92 (4.66)	0.489
**Satisfaction with genetic services**			
2–7 days	39.68 (4.13)	39.17 (4.45)	0.153
**Geriatric Depression Scale (GDS)**			
Baseline (pre‐disclosure)	0.78 (1.24)	0.80 (1.22)	0.859
2–7 day change	−0.09 (0.87)	−0.14 (1.01)	0.588
6‐week change	0.00 (1.09)	−0.10 (1.12)	0.283
6‐month change	0.47 (1.45)	0.43 (1.54)	0.775
**Impact of Genetic Testing for Alzheimer's Disease (IGT‐AD)**			
Baseline (2–7 days)	11.52 (8.19)	12.68 (9.04)	0.102
6‐week change	1.10 (6.86)	0.50 (7.04)	0.326
6‐month change	4.48 (9.54)	3.92 (9.79)	0.512
**IGT‐AD distress subscale**			
Baseline (2–7 days)	6.37 (6.53)	6.44 (7.10)	0.904
6‐week change	−1.41 (5.13)	−1.79 (5.18)	0.381
6‐month change	−1.00 (6.81)	−1.34 (7.40)	0.589
**IGT‐AD positive response subscale**			
Baseline (2–7 days)	5.15 (4.40)	6.25 (4.52)	0.003
6‐week change	2.50 (5.12)	2.30 (5.46)	0.653
6‐month change	5.48 (6.75)	5.26 (6.72)	0.717
**Perceived numerical risk of AD (0**–**100%)**			
Baseline (pre‐disclosure)	47.56 (23.01)	46.26 (21.71)	0.478
2–7 day change	−16.05 (23.97)	−13.13 (23.07)	0.136
6‐week change	−12.03 (24.23)	−10.89 (25.14)	0.587
6‐month change	−9.73 (25.14)	−9.27 (25.88)	0.845
**Perceived risk of AD relative to others (verbal scale)**			
Baseline (pre‐disclosure)	2.42 (0.99)	2.34 (1.08)	0.341
2–7 day change	−0.19 (1.08)	−0.01 (1.10)	0.059
6‐week change	−0.22 (1.12)	−0.12 (1.24)	0.354
6‐month change	−0.38 (1.32)	−0.18 (1.38)	0.104
**Perceived lifetime risk of AD (verbal scale)**			
Baseline (pre‐disclosure)	2.23 (0.88)	2.16 (0.90)	0.350
2–7 day change	−0.33 (1.05)	−0.17 (1.08)	0.079
6‐week change	−0.27 (1.13)	−0.22 (1.20)	0.642
6‐month change	−0.32 (1.24)	−0.30 (1.32)	0.836
**Perceived threat of AD**			
Baseline (pre‐disclosure)	22.11 (4.14)	21.82 (4.00)	0.385
2–7 day change	−1.02 (3.27)	−0.61 (3.54)	0.153
6‐week change	−1.08 (3.55)	−1.18 (3.76)	0.758
6‐month change	−1.36 (4.05)	−1.10 (4.12)	0.476

*Note*: All change scores are in relation to the baseline assessment.

Abbreviations: AD, Alzheimer's disease; SD, standard deviation.

^a^Primary outcomes.

Overall, 55 of 297 individuals (18.5%) in the videoconference arm experienced technical difficulties, including 18 (6.1%) that resulted in completion of *APOE* disclosure by telephone. Those who had technical difficulties had lower baseline knowledge of genetic disease (mean 37.56 [SD 4.11] among those without difficulties, mean 36.18 [SD 4.51] among those with difficulties, *p* = 0.028), lower 2–7 day satisfaction with remote counseling (mean 45.17 [SD 4.51] among those without difficulties, mean 43.56 [SD 4.91] among those with difficulties, *p* = 0.025), and lower baseline perceived risk of developing AD (mean 2.23 [SD 0.85] among those without difficulties, mean 1.87 [SD 1.04] among those with difficulties, *p* = 0.009).

In as‐treated analyses, the only statistically significant difference was the 2–7 day IGT positive subscale, which was higher (demonstrating less positive feelings) among those who used videoconferencing regardless of randomization arm (mean 6.12, [SD 4.52] among 278 using videoconferencing, 5.23 [SD 4.44] among 320 using telephone arm, *p* = 0.019). These differences did not persist at 6 weeks or 6 months.

### 3.3 Differences in cognitive and affective outcomes by arm and *APOE* genotype

In secondary analyses, we evaluated differences in outcomes between arms according to *APOE* genotype. There were no statistically significant differences at the *p* < 0.0167 level in outcomes between arms among homozygotes (Table 3). The one exception was a significant difference in change in perceived numerical risk at 2–7 days (−5.36 in telephone vs +2.78 in videoconference), although there was no difference between arms at 6 weeks and 6 months. When restricted to homozygotes who did not have prior knowledge of their *APOE* genotype (50 telephone; 51 videoconference) the results were similar.

**TABLE 3 alz71658-tbl-0003:** **Outcomes by randomized arm among *APOE* ε4 homozygotes** ^a^.

	Randomization arm	
	Telephone	Videoconference	
	(*N* = 65); Mean (SD)	(*N* = 65); Mean (SD)	*p*‐value
**Knowledge of genetic disease** ^b^			
Baseline (pre‐disclosure)	37.57 (4.73)	37.26 (3.82)	0.684
2–7 day change	1.41 (4.22)	1.19 (3.32)	0.747
6‐week change	0.70 (3.95)	1.68 (4.11)	0.185
6‐month change	0.54 (4.27)	0.75 (4.41)	0.792
**Disease‐specific distress** ^b^			
Baseline (pre‐disclosure)	4.99 (6.24)	5.02 (5.45)	0.976
2–7 day change	0.94 (4.89)	1.55 (6.61)	0.558
6‐week change	0.40 (5.23)	1.31 (5.67)	0.359
6‐month change	0.30 (4.81)	0.81 (7.09)	0.645
**Satisfaction with remote counseling** ^b^			
2–7 days	44.28 (5.08)	44.14 (4.32)	0.871
**Satisfaction with genetic services**			
2–7 days	38.24 (4.33)	37.75 (4.40)	0.532
**Geriatric Depression Scale (GDS)**			
Baseline (pre‐disclosure)	0.46 (0.77)	0.55 (0.99)	0.553
2–7 day change	0.03 (0.75)	0.09 (1.05)	0.718
6‐week change	0.16 (1.25)	0.11 (1.04)	0.792
6‐month change	0.51 (1.51)	0.29 (1.54)	0.416
**Impact of Genetic Testing for Alzheimer's Disease (IGT‐AD)**			
Baseline (2–7 day)	17.47 (8.46)	18.97 (9.77)	0.356
6‐week change	−1.34 (7.13)	−2.61 (6.45)	0.302
6‐month change	1.75 (11.21)	0.31 (11.52)	0.487
**IGT‐AD distress subscale**			
Baseline (2–7 day)	8.85 (7.94)	10.33 (8.72)	0.319
6‐week change	−2.01 (6.86)	−3.00 (5.99)	0.392
6‐month change	−1.94 (9.43)	−2.44 (10.19)	0.776
**IGT‐AD positive response subscale**			
Baseline (2–7 day)	8.62 (3.10)	8.65 (3.70)	0.969
6‐week change	0.67 (3.79)	0.39 (3.61)	0.686
6‐month change	3.69 (5.77)	2.75 (6.10)	0.407
**Perceived numerical risk of AD (0**–**100%)**			
Baseline (pre‐disclosure)	51.15 (21.62)	48.80 (20.86)	0.529
2–7 day change	−5.36 (22.04)	2.78 (18.47)	0.027
6‐week change	−1.63 (21.03)	2.47 (20.37)	0.276
6‐month change	−0.74 (22.46)	0.22 (21.78)	0.816
**Perceived risk of AD relative to others (verbal scale)**			
Baseline (pre‐disclosure)	2.66 (1.05)	2.82 (1.00)	0.394
2–7 day change	0.30 (1.00)	0.14 (1.03)	0.395
6‐week change	0.09 (0.99)	−0.03 (1.07)	0.536
6‐month change	−0.12 (1.27)	0.04 (1.25)	0.501
**Perceived lifetime risk of AD (verbal scale)**			
Baseline (pre‐disclosure)	2.43 (0.83)	2.43 (0.88)	1.000
2–7 day change	0.13 (0.84)	0.26 (0.97)	0.439
6‐week change	0.04 (0.94)	0.15 (1.07)	0.590
6‐month change	0.09 (1.01)	0.09 (1.07)	0.983
**Perceived threat of AD**			
Baseline (pre‐disclosure)	21.62 (4.38)	22.29 (3.95)	0.357
2–7 day change	−0.27 (3.08)	0.09 (2.98)	0.519
6‐week change	−0.81 (3.24)	−0.30 (3.31)	0.393
6‐month change	−1.17 (4.24)	−0.39 (3.29)	0.273

*Note*: All change scores are in relation to the baseline assessment.

Abbreviations: AD, Alzheimer's disease; SD, standard deviation.

^a^ Both first time disclosure and prior knowledge of genotype

^b^ Primary outcomes.

Among heterozygotes, there were no salient differences in knowledge or disease‐specific distress (Table 4) regardless of prior knowledge of genotype status. Among secondary outcomes at 2–7 days, the IGT‐AD positive subscale was higher (less positive feelings) in the videoconference arm. This difference did not persist at 6 weeks and 6 months. Results were similar when restricted to heterozygotes with no prior knowledge of results (137 telephone, 154 videoconference).

**TABLE 4 alz71658-tbl-0004:** Outcomes by randomized arm among heterozygotes.

	Randomization arm	
	Telephone	Videoconference	
	(*N* = 144); Mean (SD)	(*N* = 163); Mean (SD)	*p*‐value
**Knowledge of genetic disease** ^a^			
Baseline (pre‐disclosure)	37.38 (3.92)	37.17 (4.08)	0.647
2–7 day change	1.59 (3.75)	1.86 (3.77)	0.549
6‐week change	1.29 (3.74)	1.87 (4.24)	0.236
6‐month change	1.53 (4.09)	1.62 (4.45)	0.863
**Disease‐specific distress** ^a^			
Baseline (pre‐disclosure)	4.94 (5.59)	3.69 (4.86)	0.038
2–7 day change	−0.39 (5.36)	0.85 (4.92)	0.038
6‐week change	−0.19 (6.46)	0.21 (4.78)	0.559
6‐month change	0.51 (7.03)	0.88 (5.18)	0.629
**Satisfaction with remote counseling** ^a^			
2–7 days	45.02 (4.85)	45.01 (4.75)	0.987
**Satisfaction with genetic services**			
2–7 days	39.74 (4.06)	38.83 (4.52)	0.069
**Geriatric Depression Scale (GDS)**			
Baseline (pre‐disclosure)	0.75 (1.31)	0.79 (1.07)	0.764
2–7 day change	−0.13 (0.87)	−0.20 (0.87)	0.498
6‐week change	−0.05 (0.99)	−0.13 (0.97)	0.462
6‐month change	0.51 (1.42)	0.59 (1.60)	0.675
**Impact of Genetic Testing for Alzheimer's Disease (IGT‐AD)**			
Baseline (2–7 day)	11.87 (7.65)	12.78 (8.19)	0.322
6‐week change	1.21 (6.73)	0.76 (6.76)	0.590
6‐month change	4.14 (9.02)	3.59 (9.08)	0.628
**IGT‐AD distress subscale**			
Baseline (2–7 day)	6.78 (6.55)	6.19 (6.71)	0.442
6week change	−1.49 (4.86)	−1.62 (5.41)	0.830
6‐month change	−1.06 (6.44)	−1.50 (6.95)	0.596
**IGT‐AD positive response subscale**			
Baseline (2–7 day)	5.09 (4.28)	6.59 (4.47)	0.003
6‐week change	2.70 (5.07)	2.38 (5.27)	0.623
6‐month change	5.20 (6.29)	5.09 (6.48)	0.891
**Perceived numerical risk of AD (0**–**100%)**			
Baseline (pre‐disclosure)	48.13 (23.14)	45.84 (22.16)	0.381
2–7 day change	−18.38 (24.22)	−15.79 (22.16)	0.335
6‐week change	−15.21 (25.04)	−12.23 (25.46)	0.319
6‐month change	−12.50 (25.70)	−10.84 (26.17)	0.615
**Perceived risk of AD relative to others (verbal scale)**			
Baseline (pre‐disclosure)	2.35 (0.96)	2.28 (1.07)	0.501
2–7 day change	0.00 (1.01)	0.19 (1.03)	0.114
6‐week change	0.01 (1.14)	0.09 (1.25)	0.619
6‐month change	−0.26 (1.39)	−0.13 (1.45)	0.487
**Perceived lifetime risk of AD (verbal scale)**			
Baseline (pre‐disclosure)	2.22 (0.89)	2.13 (0.92)	0.415
2–7 day change	−0.40 (1.10)	−0.15 (1.04)	0.049
6‐week change	−0.21 (1.19)	−0.13 (1.23)	0.642
6‐month change	−0.31 (1.32)	−0.27 (1.37)	0.859
**Perceived threat of AD**			
Baseline (pre‐disclosure)	22.29 (4.12)	21.82 (4.06)	0.314
2–7 day change	−1.12 (3.28)	−0.81 (3.85)	0.455
6‐week change	−1.13 (3.79)	−1.40 (3.96)	0.560
6‐month change	−1.36 (3.99)	−1.25 (4.39)	0.843

*Note*: All change scores are in relation to the baseline assessment.

Abbreviations: AD, Alzheimer's disease; SD, standard deviation.

^a^ Primary outcomes.

Among non‐carriers, there were no statistically significant differences in outcomes between arms (Table 5). There were no differences between arms at the *p* < 0.0167 level when comparing any carriers (i.e., homozygous (HM) or heterozygous (HT) combined) with non‐carriers.

**TABLE 5 alz71658-tbl-0005:** Outcomes by randomized arm among non‐carriers.

	Randomization arm	
	Telephone	Videoconference	
	(*N* = 92); Mean (SD)	(*N* = 69); Mean (SD)	*p*‐value
**Knowledge of genetic disease** ^a^			
Baseline (pre‐disclosure)	37.35 (3.85)	37.64 (4.88)	0.686
2–7 day change	1.46 (3.44)	1.25 (4.20)	0.733
6‐week change	1.61 (3.52)	0.71 (4.48)	0.196
6‐month change	0.55 (3.80)	0.25 (5.27)	0.720
**Disease‐specific distress** ^a^			
Baseline (pre‐disclosure)	4.00 (5.28)	4.16 (5.59)	0.856
2–7 day change	0.71 (4.51)	0.85 (5.29)	0.860
6‐week change	−0.31 (5.44)	−0.43 (5.32)	0.886
6‐month change	0.69 (5.27)	1.02 (6.14)	0.736
**Satisfaction with remote counseling** ^a^			
2–7 days	44.30 (5.17)	45.44 (4.72)	0.166
**Satisfaction with genetic services**			
2–7 days	40.61 (3.84)	41.31 (3.51)	0.256
**Geriatric Depression Scale (GDS)**			
Baseline (pre‐disclosure)	1.04 (1.34)	1.03 (1.65)	0.961
2–7 day change	−0.13 (0.93)	−0.20 (1.25)	0.673
6‐week change	−0.04 (1.12)	−0.22 (1.48)	0.417
6‐month change	0.37 (1.47)	0.18 (1.38)	0.464
**Impact of Genetic Testing for Alzheimer's Disease (IGT‐AD)**			
Baseline (2–7 day)	6.76 (5.51)	6.55 (5.43)	0.810
6‐week change	2.64 (6.44)	2.82 (7.25)	0.874
6‐month change	6.95 (8.45)	8.13 (8.02)	0.419
**IGT‐AD distress subscale**			
Baseline (2–7 day)	3.98 (4.28)	3.35 (4.07)	0.368
6‐week change	−0.85 (3.99)	−1.06 (3.32)	0.737
6‐month change	−0.24 (4.86)	0.09 (4.72)	0.714
**IGT‐AD positive response subscale**			
Baseline (2–7 day)	2.79 (3.76)	3.19 (3.61)	0.500
6‐week change	3.49 (5.69)	3.89 (6.71)	0.704
6‐month change	7.19 (7.67)	8.05 (6.89)	0.500
**Perceived numerical risk of AD (0**–**100%)**			
Baseline (pre‐disclosure)	44.13 (23.52)	44.84 (21.52)	0.843
2–7 day change	−19.94 (22.91)	−21.86 (22.10)	0.601
6‐week change	−14.38 (23.24)	−20.29 (23.47)	0.127
6‐month change	−11.74 (24.75)	−14.53 (26.67)	0.540
**Perceived risk of AD relative to others (verbal scale)**			
Baseline (pre‐disclosure)	2.34 (0.96)	2.02 (1.02)	0.050
2–7 day change	−0.83 (0.96)	−0.64 (1.11)	0.281
6‐week change	−0.80 (0.94)	−0.70 (1.19)	0.603
6‐month change	−0.75 (1.16)	−0.50 (1.28)	0.268
**Perceived lifetime risk of AD (verbal scale)**			
Baseline (pre‐disclosure)	2.11 (0.88)	1.98 (0.84)	0.341
2–7 day change	−0.56 (1.01)	−0.64 (1.12)	0.661
6‐week change	−0.60 (1.09)	−0.79 (1.06)	0.346
6‐month change	−0.64 (1.17)	−0.72 (1.27)	0.729
**Perceived threat of AD**			
Baseline (pre‐disclosure)	22.17 (4.02)	21.38 (3.91)	0.208
2–7 day change	−1.39 (3.33)	−0.79 (3.18)	0.267
6‐week change	−1.19 (3.39)	−1.48 (3.59)	0.629
6‐month change	−1.49 (4.03)	−1.40 (4.11)	0.910

Note: All change scores are in relation to the baseline assessment.

Abbreviations: AD, Alzheimer's disease; SD, standard deviation.

^a^ Primary outcomes.

### 3.4 Moderators of patient cognitive and affective outcomes

At 2–7 days, there was no difference in satisfaction with genetic services by education status among those with fewer than 16 years of education, but those with 16+ years of education had slightly lower satisfaction in the videoconference arm (mean 39.78 [SD 4.06] in the telephone arm among those with 16+ years of education versus 39.08 [SD 4.58] in the videoconference arm, *p* = 0.021 for interaction; see Table 6). Although 2–7 day satisfaction was similar between arms among those with fewer than three relatives with dementia histories, satisfaction was increased in the video arm for those with three or more relatives (43.88 [SD 5.25] in telephone arm among those with 3+ relatives versus 45.87 [SD 4.13] in videoconference arm). Change in depressive symptoms at 2–7 days increased with age from −0.21 [SD 0.92] among 60 to 64‐year‐olds to 0.04 [SD 0.86] among 70‐ to 75‐year‐olds in the telephone arm, but decreased with age from −0.04 [SD 1.11] among 60‐ to 64‐year‐olds to –0.24 [SD 1.05] among 70‐ to 75‐year‐olds in the videoconference arm (*p* = 0.043). Change in depressive symptoms at 6 weeks showed a similar trend with age (*p* = 0.032). Non‐White participants had larger increases in knowledge at 6 weeks relative to White participants in the videoconference arm compared with less increase relative to White participants in the telephone arm (*p* = 0.039).

**TABLE 6 alz71658-tbl-0006:** Moderators of cognitive and affective outcomes.

	Telephone	Telephone	Video	Video	*p*‐value for interaction
	*N*	Mean (SD)	N	Mean (SD)	
**One**‐**week satisfaction with genetic services**					0.021
0–12 years of education	31	39.78 (4.62)	25	39.77 (3.84)	
13–15 years of education	54	39.23 (4.14)	61	39.22 (4.26)	
16+ years of education	216	39.78 (4.06)	211	39.08 (4.58)	
**One‐week satisfaction with remote counseling**					0.0499
0 FDR/SDR^a^	52	44.33 (5.72)	67	44.22 (4.93)	
1–2 FDR/SDR	146	45.28 (4.46)	127	44.52 (4.83)	
3+ FDR/SDR	103	43.88 (5.25)	103	45.87 (4.13)	
**Change from baseline** ^b^: **1**‐**week Geriatric Depression Scale (GDS)**					0.043
60–64 years	84	−0.21 (0.92)	65	−0.04 (1.11)	
65–69 years	105	−0.14 (0.82)	131	−0.10 (0.93)	
70–75 years	112	0.04 (0.86)	101	−0.24 (1.05)	
**Change from baseline** ^b^: **6**‐**week Geriatric Depression Scale (GDS)**					0.032
60–64 years	84	−0.22 (1.10)	65	0.08 (1.09)	
65–69 years	105	0.08 (0.86)	131	−0.10 (1.18)	
70–75 years	112	0.09 (1.24)	101	−0.21 (1.06)	
**Change from baseline** ^b^: **6**‐**week knowledge of genetic disease**					0.039
White	280	1.30 (3.68)	277	1.40 (4.25)	
Non‐White/Missing	21	0.73 (4.41)	20	3.70 (4.24)	

Abbreviation: SD, standard deviation.

^a^ FDR/SDR = first‐degree relative/second‐degree relative.

^b^ Baseline is defined as pre‐disclosure.

## 4 DISCUSSION

To our knowledge, this represents the largest longitudinal randomized prospective study comparing the disclosure of genetic results—and specifically *APOE* genotype information—by telephone as compared to real‐time videoconference and including robust patient reported outcomes. This is particularly relevant given the growing clinical importance of *APOE* genotype in the context of recently approved amyloid‐modifying therapies.^3^ We did not find that videoconference was associated with significantly improved patient reported outcomes as compared to telephone disclosure, and conclude that both telephone and videoconference modalities are appropriate and effective for disclosing *APOE* results to cognitively unimpaired individuals 60‐ to 75‐years‐old regardless of genotype. No significant differences were observed between groups in terms of knowledge gain or affective responses. This held true even among participants receiving positive results associated with an increased risk of AD, including both *APOE* ε4 homozygotes and heterozygotes. Heterozygotes randomized to telephone reported significantly more positive impacts of genetic testing than those randomized to videoconference at 2–7 days, but this difference was transient and did not persist over time.

*APOE* testing is now routinely performed in patients with MCI and mild dementia due to AD who are being considered for anti‐amyloid therapies, and as more *APOE* ε4 carriers are identified, testing among patients’ first‐degree relatives is also expected to increase.^2^ As such, understanding patient outcomes following the disclosure of *APOE* results and identifying optimal methods for communicating this information is increasingly important. This study suggests that *APOE* result disclosure in cognitively unimpaired patients, when conducted by a GC by either telephone or videoconference, is a reasonable approach with no clinically significant or persistent differences in patient outcomes between modalities. These findings are particularly relevant in the context of limited access to genetic specialists, highlighting the value of remote delivery models in expanding access to equitable care. It is important to note, however, that these results were obtained in the context of structured genetic counseling; outcomes may differ when testing occurs without formal counseling. Given the scarcity of real‐world data on clinical *APOE* testing for AD susceptibility, referral for genetic counseling may be advisable (e.g., in relatives or others who are cognitively unimpaired and not undergoing testing for treatment indications). In this study, centralized genetic counseling with multi‐state licensure allowed nationwide access.

A persistent challenge in the broader implementation of genetic services—including *APOE* testing—is the limited reimbursement for genetic counseling. Additional policy advocacy is needed to ensure coverage of genetic counseling services in general, and when delivered remotely, as part of patient‐centered care.^38^ Although telehealth reimbursement expanded during the coronavirus disease 2019 (COVID‐19) pandemic, continued support is essential to sustain innovative delivery models and fully realize the promise of precision medicine to improve medical care.^39^

Notably, this study utilized a one‐visit model in which an educational video and brochure were utilized to introduce key educational and counseling topics, and the single genetic counseling session encompassed elements of both pre‐test and disclosure.^12^ The favorable patient‐reported outcomes observed suggest that a single‐visit approach—comprising pre‐test video education followed by result disclosure with a GC—is a reasonable and effective alternative to the traditional two‐visit genetic counseling model. In the context of limited reimbursement and increasing demand for genetic testing across medical disciplines, such streamlined models have the potential to enhance access to genetic services and reduce overall counseling costs while maintaining the involvement of a genetic specialist. It is important to emphasize that these outcomes were achieved with GC‐led result disclosure; outcomes may differ if results are delivered electronically via the electronic medical record, particularly in light of the 21st Century Cures Act, which facilitates direct result release without accompanying counseling or patient‐centered educational materials.^40^ Similarly, outcomes could differ with disclosure of results by other medical providers, as identified in REVEAL3, where non‐inferiority of telephone counseling was met for participants seen by a GC but not for those who received counseling with other medical providers.^6^ Moreover, it remains unclear whether similar outcomes would be observed without the use of the pre‐test materials employed. Emerging models of education, including self‐directed digital tools, have shown promise in other areas of genetic medicine^41^, ^42^ and are currently being evaluated for *APOE* result disclosure in the ongoing eSMARTER study (NCT06459583).^43^

In secondary analyses, we identified differences in patient‐reported outcomes by years of education, age, race/ethnicity, and number of relatives with a history of AD. Individuals with 16+ years of formal education—typically corresponding to a college degree or higher—reported greater satisfaction with telephone disclosure, whereas no modality‐related differences were observed among those with less than 16 years of education. Older participants, particularly those over age 70, appeared to experience less depressive symptoms following disclosure via videoconference, and non‐White participants demonstrated greater knowledge gains with videoconference compared to telephone. These differences in knowledge gain may be influenced by unmeasured factors such as health or genetic literacy, which were not assessed in this study. Few studies evaluating different delivery modalities have evaluated moderators, although a meta‐analysis of telepsychology in veterans reported that video is more effective than phone for patients with depression or trauma.^44^ Further research evaluating subgroups who benefit more or less from videoconference could help inform recommendations for *APOE* result disclosure in real‐world clinical settings. GCs may wish to prioritize videoconference disclosure for patients with depression or strong family history of dementia, for those from racial and ethnic minority groups, or when lower genetic knowledge or lower health literacy is suspected. Additional research in these populations is warranted to refine disclosure strategies and optimize outcomes related to the return of *APOE* results.

This study has several limitations. The study was terminated early and did not reach original planned enrollment. All disclosures were conducted by GCs, and it remains unclear whether similar outcomes would be observed with other healthcare providers, particularly as result disclosure by non‐genetics professionals becomes more common in clinical practice. GS1 screening protocols limited the study population to cognitively unimpaired individuals without moderate to severe symptoms of depression or anxiety, thus these results may not generalize to typical clinic populations nor individuals with MCI, who are often candidates for *APOE* testing in the context of anti‐amyloid therapies. The study was conducted prior to the COVID‐19 pandemic, and delays in data reporting due to General Data Protection Regulation (GDPR) regulations mean that participant experiences may not fully reflect the current landscape and increased adoption of videoconferencing during the pandemic. Technology challenges that were encountered by some patients in the videoconference arm that may have contributed to the small differences between arms are likely less frequent with current videoconference platforms. The participants in this study had a high mean level of education (16.5 years) and may represent an early‐adopter population with a strong interest in dementia prevention, both of which could limit generalizability. Further research is needed in more diverse and representative clinical populations, including individuals with varying backgrounds, racial and ethnic identities, and levels of health literacy. This is also the largest study comparing videoconference to telephone disclosure of genetic testing, although it is unclear whether findings would be similar in other disease types (e.g., cancer or cardiovascular disease).

In summary, both telephone and videoconference are viable methods for disclosing *APOE* results to cognitively unimpaired individuals across all genotypes. Although overall outcomes were comparable between modalities, secondary analyses suggest that videoconference disclosure may offer modest benefits for certain subgroups, including reductions in depressive symptoms among individuals over 70 years of age and greater knowledge gains among non‐White participants.

## CONFLICT OF INTEREST STATEMENT

E. Wood, B. Egleston, D. Ofidis, C. Cacioppo, C. Langlois, M. Weinberg, S.Y.H Kim, and J.S. Roberts have no interests to declare. J. Langbaum has received consulting fees from Biogen and Denovo Biopharma. E. Reiman is a cofounder and advisor to AlzPath, and is a compensated advisor to Alzheon, Cognition Therapeutics, Denali Therapeutics, Enigma, Jocanta, Retromer Therapeutics, and Vaxxinity. P. Tariot has received funding support from Eli Lilly, and received consulting fees from AbbVie, Acadia Pharmaceuticals, AC Immune, Athira Pharma, Axsome Therapeutics, Bristol Myers Squibb, Cognition Therapeutics, Cognito, Corium, CuraSen Therapeutics, Eisai, Genentech, ImmunoBrain, Janssen, Lundbeck, MapLight, Merck & Co., Novartis, Novo Nordisk, ONO Pharma, Otsuka/Astex Pharmaceuticals, Roche, and T3D Therapeutics. J. Karlawish has received consulting fees from Darmiyan and Linus Health. A. Bradbury receives partial research funding from Astrazeneca for a cancer care delivery study. Author disclosures are available in the Supporting Information.

## CONSENT STATEMENT

The Remote COmmuNication of GeNEtiC Test Results for *APOE* (CONNECT4) study was approved by the University of Pennsylvania Institutional Review Board. Informed consent was obtained from all participants, and all participant data were de‐identified.

## Supporting information




**Supporting Information**: alz71658‐supp‐0001‐Figure S1.pdf

**Supporting** Information: alz71658‐supp‐0002‐Table S1.docx

**Supporting Information**: alz71658‐supp‐0003‐ICMJE.pdf

**Supporting Information**: alz71658‐supp‐0004‐SuppMat.docx
