# Supplementary material for: CONNECT4 APOE: A randomized trial of telephone versus real‐time two‐way videoconference for disclosure of APOE genotype results in cognitively unimpaired adults
Source: Alzheimers Dement. 2026 Jul 27;22(7):e71658. doi: 10.1002/alz.71658 (PMC13408020; doi:10.1002/alz.71658)
Supplement: Supplementary file 1 — Supporting Information: alz71658‐supp‐0001‐Figure S1.pdf [file ALZ-22-e71658-s003.pdf]

APOE TELEGENETICS COUNSELING CHECKLIST SHORT FORM – ALL DISCLOSURES

Date: ______ Subject ID: _________________ Genetic Counselor initials: _______ Site PI:____________________

Start Time (participant/GC in visit room): __________am/pm End Time:________am/pm

Session Type: ☐ CONNECT4 ☐ GDGS **Consented to CONNECT4:** ☐ Y ☐N ☐ N/A

Consented to Record: ☐ Y ☐N **Source of report for disclosure:** ☐ICF#1 ☐Genematch ☐Other CLIA report

Race: ☐Asian ☐American Indian/Alaskan Native ☐Caucasian ☐ Unknown/Not Reported
☐Black/African American ☐Native Hawaiian or Pacific Islander ☐More Than One Race

Ethnicity: ☐Hispanic or Latino ☐Not Hispanic or Latino ☐Unknown or Not Reported

1. INTRO

☐ Review participant intake form with site coordinator only

☐ Introductions

Participant's friends / relatives:________________________

☐ Confirm all TELEMEDICINE participants can hear and/or see adequately

Address possible glitches, camera position, speech delay, speaker phone use, taking notes

Confirm visual aid packet is available (note: not all slides will be reviewed)

Confirm participant is aware of recording

2. CONTRACTING

☐ Affirm session purpose and verify participant is ready to start counseling and still interested in participating

☐ Emotional Probe (How are you feeling about this?) ☐ Knowledge Probe (Are there any particular questions/concerns?)

Does the participant know their APOE result? **Yes**_____ **No**_____

3. PREVIOUS APOE RESULT DISCLOSURE PARTICIPANTS ONLY *(GC to "X" thru boxed section if not relevant)*

Source of patient result: ☐ 23 & Me ☐ CLIA Lab ☐ Other_______

APOE RESULT (per participant): _________________)

Assess how long participant has known results:_____________

Briefly review history of results disclosure (ie, who disclosed, DTC testing, etc…)

☐ Emotional Probe (How do you feel about this?) ☐ Knowledge Probe (Any particular questions/concerns about your results?)

ALL PARTICIPANTS:

4. PERSONAL AND FAMILY HISTORY

☐ Attention to AD/dementia in family history

5. EDUCATION

☐ Diagnosis definitions: Aging normally, MCI, Dementia and Alzheimer's disease

☐ APOE risk factor

☐ No medical prevention options

☐ Review possible results

6. RESULT IMPLICATIONS/IMPACT

☐ Inheritance

☐ Considerations of genetic testing

☐Personal/psychological

☐Family

☐ Confidentiality/Genetic Discrimination/GINA

☐ Emotional Probe (How are you feeling about this?) ☐ Knowledge Probe (Any questions?)

7. FIRST TIME DISCLOSURE PARTICIPANTS ONLY

☐ **Assess if participant is INTERESTED in learning APOE test results**

☐ YES (DISCLOSE APOE RESULT and AD risk estimate: ________________________________)

☐ NO (Reason: ____)

☐ Emotional Probe (How are you feeling about this?) ☐ Knowledge Probe (Would you like me to review any of the earlier information now that we know your result?)

ALL PARTICIPANTS:

9. ☐ MODIFIERS AND HEALTHY BRAIN AGING

10.☐ TEACH BACK

Clarify any wrong information/misunderstandings

11. CLOSING ☐ Invite SC back into room, recap main points in the session, review next steps

APOE TELEGENETICS COUNSELING CHECKLIST SHORT FORM – ALL DISCLOSURES

Technology Evaluation Post Session

Were there any technology issues/troubleshooting while initiating the session? ☐ **Yes** ☐ **No**
If Yes, please note how long issues persisted: __________ (minutes)
Was the session recorded? ☐ **Yes** ☐ **No**
What modality was used for the disclosure? ☐ **Videoconference** ☐ **Telephone**

Videoconference Technology Evaluation:

What Videoconference platform was used?

☐ **BlueJeans** ☐ **Medisprout** ☐ **Other _______**

What was the status of the session?

☐ **COMPLETED:** (defined as few to no "glitches" with negligible impact on communication).
☐ **COMPLETED WITH DISRUPTIONS:** (defined as moderate "glitches" causing communication difficulty, including: screen freeze, video/audio lag, feedback).
☐ **FAILURE:** (defined as excessive "glitches" causing severe impact on communication leading to termination of the session).

Was a secondary platform used? ☐ **Yes** ☐ **No**

If Yes:
What was the secondary platform used?

☐ **BlueJeans** ☐ **Medisprout** ☐ **Other________**

What was the status of the session for the secondary platform?

☐ **COMPLETED**
☐ **COMPLETED WITH DISRUPTIONS**
☐ **FAILURE**

Phone Technology Evaluation:

What was the status of the session?

☐ **COMPLETED:** (defined as few to no "glitches" with negligible impact on communication).
☐ **COMPLETED WITH DISRUPTIONS:** (defined as moderate "glitches" causing communication difficulty, including: static, echoing, or dropped call).
☐ **FAILURE:** (defined as excessive "glitches" causing severe impact on communication leading to termination of the session).

All Modalities:
If technology issues were noted:

Who experienced technology issues?
☐ **Penn Site** ☐ **Partner Site**
☐ **Both Sites** ☐ **Unable to Determine**

Who was involved in troubleshooting technology issues? (multiple responses may be noted)
☐ **GC** ☐ **Penn Research Staff**
☐ **Partner Site Staff** ☐ **Patient**
☐ **N/A**

Additional notes about session: intended to capture unanticipated challenges occurring outside of set technology modality, (i.e., equipment failures or challenges, such as charger unplugged and battery died, computer rebooting required mid-session, etc...)
____
____
____
____
____
