# Supplementary material for: CONNECT4 APOE: A randomized trial of telephone versus real‐time two‐way videoconference for disclosure of APOE genotype results in cognitively unimpaired adults
Source: Alzheimers Dement. 2026 Jul 27;22(7):e71658. doi: 10.1002/alz.71658 (PMC13408020; doi:10.1002/alz.71658)
Supplement: Supplementary file 2 — Supporting Information: alz71658‐supp‐0002‐Table S1.docx [file ALZ-22-e71658-s004.docx]

**Supplemental Table 1. Content of Generation Study Pre-Disclosure Educational Video**

| Section | Outline of content covered | Time |
| --- | --- | --- |
| Overview of Alzheimer’s Disease and *APOE* gene | - Explanation of the terms   “Alzheimer’s disease” (AD), “dementia,” and “mild cognitive impairment”   - Introduction to the *APOE* gene - The types of *APOE* and possible genotypes - Definition of “risk factor” - Examples of non-genetic risk factors for AD - Role of *APOE* e4 as a risk factor for AD - Lifetime risks of AD based on *APOE* genotype | 5 min 0 sec |
| One question to check understanding | What form of *APOE* increases risk for AD? | Variable |
| Considerations of Learning *APOE* Results | - Possible positive and negative emotional impacts of learning *APOE* result - Possible impact on family members, including genetic inheritance and family communication about AD risk - Possible impact on future planning - Genetic Information Nondiscrimination Act | 4 min 42 sec |
| Two questions to check understanding | How does having the *APOE* e4 gene affect the chances that someone will get AD? Could I become more worried or anxious if my *APOE* test results suggest an elevated risk for developing AD? | Variable |
| Summary | Reminder of key points, including:   - Role of *APOE* results in Generation Study eligibility - *APOE* e4 as risk factor for AD - It is a personal decision to learn *APOE* results | 1 min 31 sec |
|  |  | Total time: 11 min 13 sec |

In addition to the educational content, a total of three questions were spaced between the sections. These questions were designed to reinforce key educational points. The correct answer with an explanation was provided to all participants, regardless of whether they answered the item correctly. A correct answer was not required to move forward with the educational content. As the questions were placed between video sections and were untimed, they would have added a variable amount of time to complete the educational video content.
