# Supplementary material for: CONNECT4 APOE: A randomized trial of telephone versus real‐time two‐way videoconference for disclosure of APOE genotype results in cognitively unimpaired adults
Source: Alzheimers Dement. 2026 Jul 27;22(7):e71658. doi: 10.1002/alz.71658 (PMC13408020; doi:10.1002/alz.71658)
Supplement: Supplementary file 4 — Supporting Information: alz71658‐supp‐0004‐SuppMat.docx [file ALZ-22-e71658-s002.docx]

**Statistical considerations and analyses**

**Hypothesis 1.a.:** We hypothesize that patients randomized to remote two-way videoconferencing will have a) greater increase in genetic knowledge, b) greater decreases in disease-specific distress and c) greater satisfaction post genotype disclosure.

For the primary analysis, we will assess outcomes a-c at 2 to 7 days after baseline in the two arms. We will create change scores to investigate increases in a) genetic knowledge, b) disease-specific distress, and c) satisfaction between baseline and 2 to 7 days. We will use t-tests to compare change scores between treatment arms. For the primary analyses, we will use an intention to treat approach whereby comparisons are made between randomization arms rather than between as-treated arms. In secondary analyses, we will examine differences between the as treated groups and per-protocol groups.

***Sample Size Justification for Aim 1:*** We chose 1500 persons per arm (3,000 total) so that we would have sufficient power to detect our 3 primary associations of interest after any participant losses to follow-up. This assumes that the sample size with evaluable outcomes will be reduced by up to 30% (450 per arm or 900 total) by loss to follow-up, providing approximately 1050/arm for evaluation of our 3 primary outcomes. If recruitment is lower than expected, we will have sufficient power to detect differences in knowledge and satisfaction with 398 evaluable subjects/arm.

The primary analysis will be intention to treat t-tests assuming unequal variances of the baseline to 2-7 day change scores for the continuous variables. In order to determine anticipated power, we used preliminary estimates from the published Risk Evaluation and Education for Alzheimer’s Disease (REVEAL) study for IES disease-specific distress[^12^](#_ENREF_12) and our COGENT trial for knowledge and satisfaction (see table below).

For the primary analyses, we will use an intention-to-treat approach in which we compare change score differences between randomization arms rather than between the as-treated groups. In secondary analyses, we will examine per-protocol and as-treated effects. In calculating the number we need to detect statistically significant differences in the primary intention-to-treat analyses, we set the power to 85% and the Type I error rate to 1.67% (2-sided). We set the Type I error rate to 1.67% by using a Bonferroni correction with three comparisons on a 5% family-wise Type I error rate (5%/3=1.67%). We see from below that we have excellent power to detect differences for knowledge and satisfaction even if the study only recruits 398 per arm (796 total). For disease-specific distress, we have excellent power if we have 1,036/arm, which is well below the target of 1,500 (and also below the potential evaluable target of 1,050 participants/arm). We used PASS-11 software for the power calculations.

| **Sample size estimates for 85% power, 1.67% Type I error (2-sided). SD=standard deviation** | | |
| --- | --- | --- |
| Variable | Observed estimates  Mean (SD) group 1 vs group2 | Number needed to detect difference |
| Knowledge (using COGENT change score estimates) | .20 (2.64) vs. -.48 (2.54) | 342 / arm = 684 total |
| Disease –specific distress (IES total, using REVEAL 6-week cross-sectional estimates) | 6.7 (10.0) vs. 5.2 (9.9) | 1,036 / arm = 2,072 total |
| Satisfaction (using COGENT change score estimates) | 0.79 (4.96) vs. 2.00 (4.99) | 398 /arm = 796 total |

**Hypothesis 1.b.:** As suggested by our theoretical model, related literature and preliminary data, we hypothesize that participant factors (e.g. personal or family history, demographics), will moderate the impact of remote communication modality (videoconferencing v. telephone communication) on the short-term cognitive and affective responses to remote disclosure.

We will examine moderators by investigating multiple linear regressions of the cognitive and affective change score responses in which we include randomization arm indicator, moderator, and interaction between randomization arm indicator and moderator as covariates into the model. An interaction is created by multiplying the randomization arm indicator (0/1 variable) by the moderator variable. This aim is exploratory and hypothesis generating only. We seek to collect preliminary evidence regarding possible subgroups for which remote videoconference communication is particularly useful as compared to telephone communication of genotype result to better understand policies for implementation of remote genetic services in the era or Precision Medicine. To increase the power to detect potential moderators, we will examine models in which we include moderators separately. We will also investigate moderators of the as-treated groups per-protocol groups similarly.

**Hypothesis 2.a:** We hypothesize that two-way videoconferencing will be associated with greater longitudinal increases (or less decline) in genetic knowledge and test recall and longitudinal distress (e.g., disease-specific distress). Our primary short-term outcomes for Aim 2a include change in a) knowledge, b) test recall, c) disease-specific distress. Secondary outcomes will include depression, positive and negative responses to testing and uncertainty.

We will use longitudinal growth curve models to investigate longitudinal outcomes. Specifically, we will use multiple linear regressions for the continuous knowledge, distress, and behavioral outcomes and multiple logistic regressions for the binary test recall outcome. We will estimate the regressions by Generalized Estimating Equations with appropriate working correlation matrices to account for within subject correlation of repeated measures. As covariates in the models, we will include time dummy indicators (0/1 binary variables for each post-baseline survey wave, leaving the baseline as a reference category), as well as an indicator variable for randomization arm, and the interaction between the time indicators and randomization arm indicator (an interaction is created by multiplying two variables). We will also repeat the analysis but instead of randomization arm, assign individuals to their as-treated groups and again to their per-protocol groups.

**Hypothesis 2.b**: Participant factors (e.g., family history, demographics, perception of risk) will moderate the impact of remote communication modality (videoconferencing v. telephone communication) on longitudinal cognitive and affective responses to remote disclosure.

We will include the family history, demographic variables, and perception of risk variables as covariates in the growth curve models described in hypothesis 2.a, as well as the interaction terms between these variables and randomization arm. We will investigate a full model with all of the variables and interactions and also models with each variable and interaction term separately (i.e., reduced and full models). Finally, we will examine models that include all of the three-way interactions between the participant factors, randomization arm, and time indicators; these three-way interaction models will include the two way interactions as well. We will also investigate effects after assigning participants to their as-treated groups and per protocol groups.

***Missing data:*** We do not expect there to be substantial missing baseline data, although there may be loss to follow-up over time. In secondary analyses, we will account for missing baseline data using the multiple imputation technique of Raghunathan and colleagues with 100 imputed datasets (reference: Raghunathan TE, Lepkowski JM, Van Hoewyk J, Solenberger P. A multivariate technique for multiplying imputing missing values using a sequence of regression models. Survey Methodology. 2001;27:85-95). We will contrast the results obtained through imputation and those obtained from complete case analyses to investigate if missing data bias could be substantially affecting our inferences. We will not use multiple imputation for loss to follow-up or missing outcome responses. We will simply exclude those with missing outcome data from the relevant analyses.
